# Automatic Detection of Prostate Tumor Habitats using Diffusion MRI

**DOI:** 10.1038/s41598-018-34916-4

**Published:** 2018-11-14

**Authors:** Yohann Tschudi, Alan Pollack, Sanoj Punnen, John C. Ford, Yu-Cherng Chang, Nachiketh Soodana-Prakash, Adrian L. Breto, Deukwoo Kwon, Felipe Munera, Matthew C. Abramowitz, Oleksandr N. Kryvenko, Radka Stoyanova

**Affiliations:** 1 Department of Radiation Oncology, University of Miami Miller School of Medicine, Miami, FL USA; 2 Department of Urology, University of Miami Miller School of Medicine, Miami, FL USA; 3 University of Miami Miller School of Medicine, Miami, FL USA; 4 Biostatistics and Bioinformatics Shared Resources, Sylvester Comprehensive Cancer Center, University of Miami Miller School of Medicine, Miami, FL USA; 5 Department of Radiology, University of Miami Miller School of Medicine, Miami, FL USA; 6 Department of Pathology and Laboratory Medicine, University of Miami Miller School of Medicine, Miami, FL USA

## Abstract

A procedure for identification of optimal Apparent Diffusion Coefficient (ADC) thresholds for automatic delineation of prostatic lesions with restricted diffusion at differing risk for cancer was developed. The relationship between *the size of* the identified Volumes of Interest (VOIs) and Gleason Score (GS) was evaluated. Patients with multiparametric (mp)MRI, acquired prior to radical prostatectomy (RP) (n = 18), mpMRI-ultrasound fused (MRI-US) (n = 21) or template biopsies (n = 139) were analyzed. A search algorithm, spanning ADC thresholds in 50 µm^2^/s increments, determined VOIs that were matched to RP tumor nodules. Three ADC thresholds for both peripheral zone (PZ) and transition zone (TZ) were identified for estimation of VOIs at low, intermediate, and high risk of prostate cancer. The determined ADC thresholds for low, intermediate and high risk in PZ/TZ were: 900/800; 1100/850; and 1300/1050 µm^2^/s. The correlation coefficients between the size of the high/intermediate/low risk VOIs and GS in the three cohorts were 0.771/0.778/0.369, 0.561/0.457/0.355 and 0.423/0.441/0.36 (p < 0.05). Low risk VOIs mapped all RP lesions; area under the curve (AUC) for intermediate risk VOIs to discriminate GS6 vs GS ≥ 7 was 0.852; for high risk VOIs to discriminate GS6,7 vs GS ≥ 8 was 0.952. In conclusion, the automatically delineated volumes in the prostate with restricted diffusion were found to strongly correlate with cancer aggressiveness.

## Introduction

The use of multiparametric MRI (mpMRI) is rapidly gaining momentum in the management of prostate cancer because of its improved diagnostic potential. Diffusion Weighted Imaging (DWI) and the associated Apparent Diffusion Coefficient (ADC) maps play a central role in the Prostate Imaging Reporting and Data System (PI-RADS)^1,2^, as well as in computer-aided diagnosis (CAD) systems and in the search for quantitative imaging biomarkers for tumor assessment^3–7^. Mean ADC values or other parameters of the ADC distribution within suspect cancer areas are typically investigated in relation to cancer aggressiveness. However, the ***size*** of the prostatic ***3D*** regions of decreased diffusion have not been considered, partly due to the fact that setting a threshold below which to determine these volumes of “low” ADC, a.k.a Volumes of Interest (VOIs), is not trivial. In previous work, ADC = 800 µm^2^/s and 850 µm^2^/s were used as such thresholds in the Peripheral (PZ) and Transition Zone (TZ), correspondingly^8^. These ad-hoc values were selected based on literature and empirical observations^9–14^. In a subsequent pilot study of a retrospective patient cohort (n = 139), the ***size*** of the VOIs, defined by pixels with ADC < 800/850 µm^2^/s (PZ/TZ) were significantly correlated with biopsy Gleason Score (GS)^15^.

These preliminary results motivated the current study for systematic determination of optimal ADC thresholds, associated with high, intermediate and low risk of cancer. The underlying hypothesis is that the size of the VOIs with reduced diffusion are related to tumor aggressiveness. The purposes of this study are: *(i)* to develop a procedure for identification of optimal ADC thresholds for automatic delineation of prostatic lesions at differing risk for cancer; and *(ii)* to evaluate the relationship between the size of these VOIs and GS. The ADC thresholds were determined in prostatectomy samples, using histopathology location and aggressiveness of the tumor nodules as “ground truth”. The size of the VOIs, defined by these thresholds, were evaluated for correlation with GS in independent datasets.

## Results

### Patients

An Institutional Review Board (IRB) at the University of Miami approved a protocol for retrospective review of prostate mpMRI. The IRB waived the need for informed consent. All research was performed in accordance with the relevant guidelines/regulations associated with the protocol. Imaging datasets from three cohorts of patients were analyzed: *(i)* eighteen patients, who underwent radical prostatectomy (RP); *(ii)* 21 patients, who underwent MRI/Ultrasound fused (MRI-US) biopsy; and *(iii)* 139 patients with template transrectal ultrasound-guided (TRUS) biopsies. The inclusion criteria was that mpMRI was acquired on the 3 T Discovery MR750 magnet (GE, Waukesha, WI). Here and throughout the manuscript, these datasets are referred as *prostatectomy*, *target biopsies*, and *template biopsy datasets*. The clinical characteristics of the patients are presented in Table 1. mpMRI were acquired between May 2012 and November 2016. Surgery/biopsies were carried out (mean ± SD) 74 ± 45/48 ± 56 days after imaging.

**Table 1:** Patients characteristics and Gleason Score distributions in analyzed datasets.

	Prostatectomy dataset	Targeted Biopsy dataset	Template Biopsy dataset
**Number of patients**	18	21	139
**Age (years)**			
Mean	64.72	67.8	69.31
Median	64	68	69
Range	60–72	52–79	48–89
**PSA (ng/ml)**			
Mean	10.5	11.85	6.85
Median	7	9.8	12.3
Range	2.6–33.6	2.5–31.46	0.5–226
T-stage*			
T1	0	8	75
T2	11	8	46
T3	7	5	18
**Number of lesions**	53	28	NA^**^
PZ	28	23	91^†^
TZ	25	5	47^†^
**Gleason Score**			
6	26	7	35
7 (3 + 4)	18	11	46
7 (4 + 3)	0	4	28
8 (4 + 4)	1	3	13
9 (4 + 5)	6	3	12
9 (5 + 4)	1	0	2
10	1	0	3

*Abbreviations:* PSA = Prostate Specific Antigen; PZ = Peripheral Zone; TZ = Transition Zone.

^*^Pathological stage for prostatectomy patients; clinical stage for biopsy cohorts.

^**^Highest Gleason Score from the biopsy session is used.

^†^Estimated location, based on the largest detected lesion (see text for details). Note that one lesion (GS 6) was not detected.

### Prostatectomy dataset

#### Mapping tumor lesions from RP on MRI

**Figure 1:**
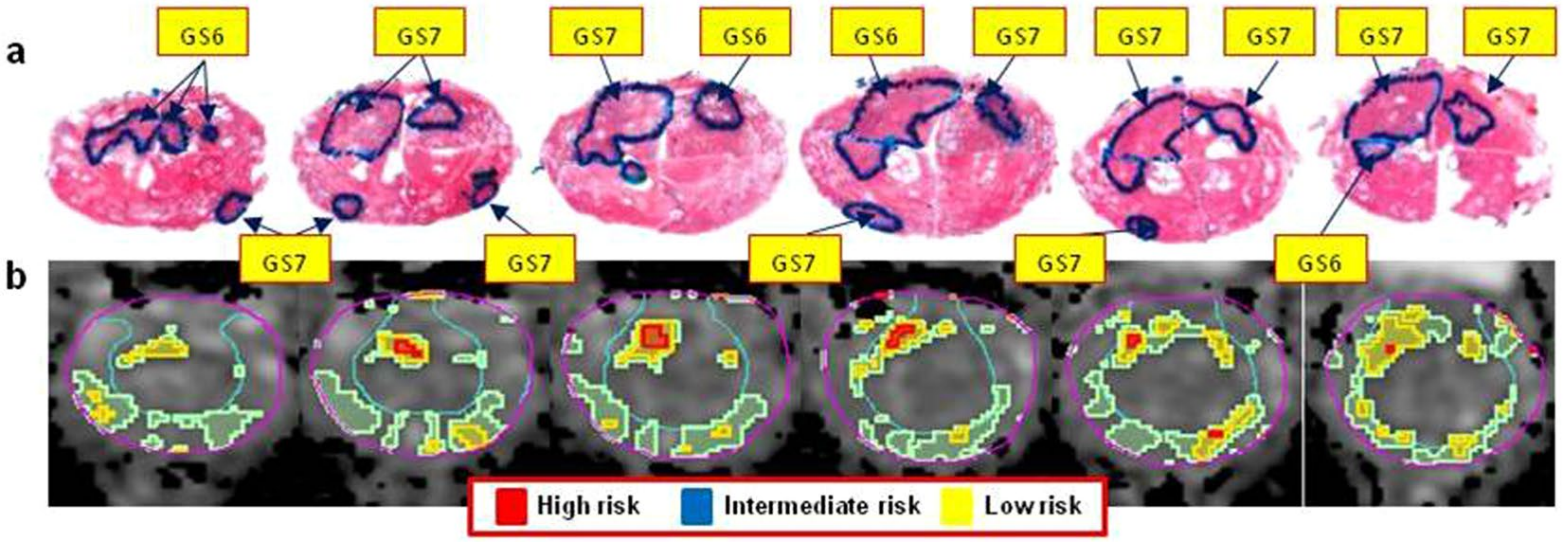
Correlation of histopathology with identified ADC areas of cancer. (**a**) Pseudo-whole mount H&E-stained histopathology sections. Consecutive axial slices are displayed from apex (left) to base (right). Tumor nodules are marked and labeled by a pathologist; (**b**) Corresponding transverse slices of ADC and automatically created regions based on ADC thresholds for high (red), intermediate (blue) and low (yellow) risk. ADC thresholds for PZ and TZ, respectively are: high risk - 900 µm^2^/s and 800 µm^2^/s; intermediate risk: 1100 µm^2^/s and 850 µm^2^/s; low risk - 1300 µm^2^/s and 1050 µm^2^/s.

**Figure 2:**
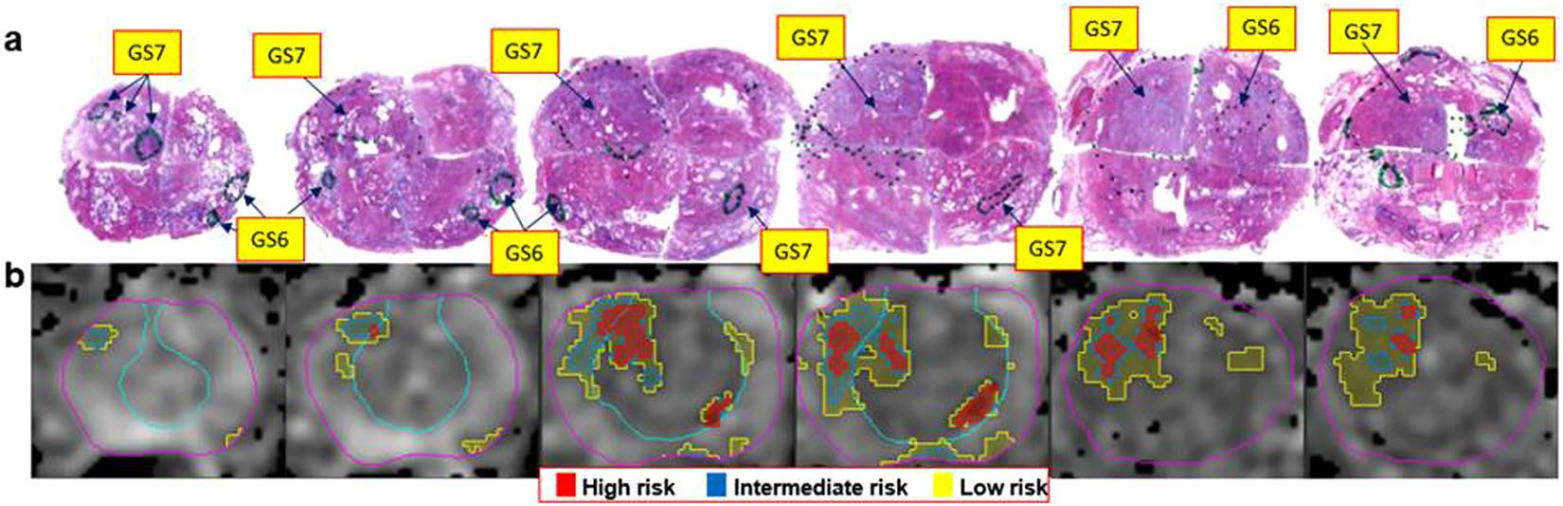
Correlation of histopathology with identified ADC areas of cancer. (**a**) Pseudo-whole mount H&E-stained histopathology sections. Consecutive axial slices are displayed from apex (left) to base (right). Tumor nodules are marked and labeled by a pathologist; (**b**) Corresponding transverse slices of ADC and automatically created regions based on ADC thresholds for high (red), intermediate (blue) and low (yellow) risk. ADC thresholds for PZ and TZ, respectively are: high risk - 900 µm^2^/s and 800 µm^2^/s; intermediate risk: 1100 µm^2^/s and 850 µm^2^/s; low risk - 1300 µm^2^/s and 1050 µm^2^/s. Note the presence of high risk regions only in Gleason Score (GS) 7 lesions.

RP specimens were handled in accordance with well-established procedures^16^. All specimens were reviewed by an urologic pathologist (ONK) who was blinded to the imaging findings. Cancer was mapped on hematoxylin and eosin (H&E) stained slides (histopathology ROIs). Each tumor nodule was individually staged and graded according to the latest recommendations^17,18^. The annotated slides were scanned and prostate quadrants were “stitched” into a pseudo whole-mount RP sample (Figs 1a and 2a).

**Figure 3:**
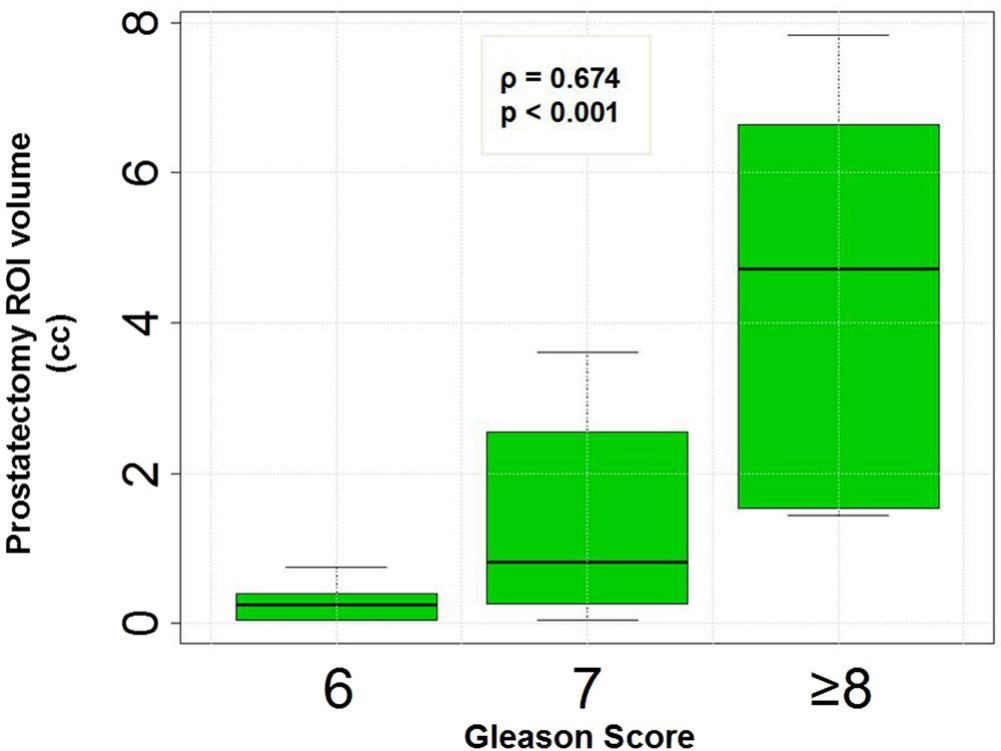
Box plots of radical prostatectomy tumor volumes versus Gleason Score.

The locations of the histopathology Regions of Interest (ROIs) from radical prostatectomy were mapped onto MRI (rpROI) in MIM (Cleveland, OH). Out of a total of 53 H&E nodules (Table 1), three regions could not be mapped on MRI. The rpROI volumes were measured in MIM and summarized in Supplementary Table S1, together with their location (PZ/TZ) and GS. In Fig. 3, the box plot of the rpROI volumes versus GS is presented (ρ = 0.674, p < 0.001).

#### ADC thresholds for identifying prostate volumes at differing risk for cancer

**Figure 4:**
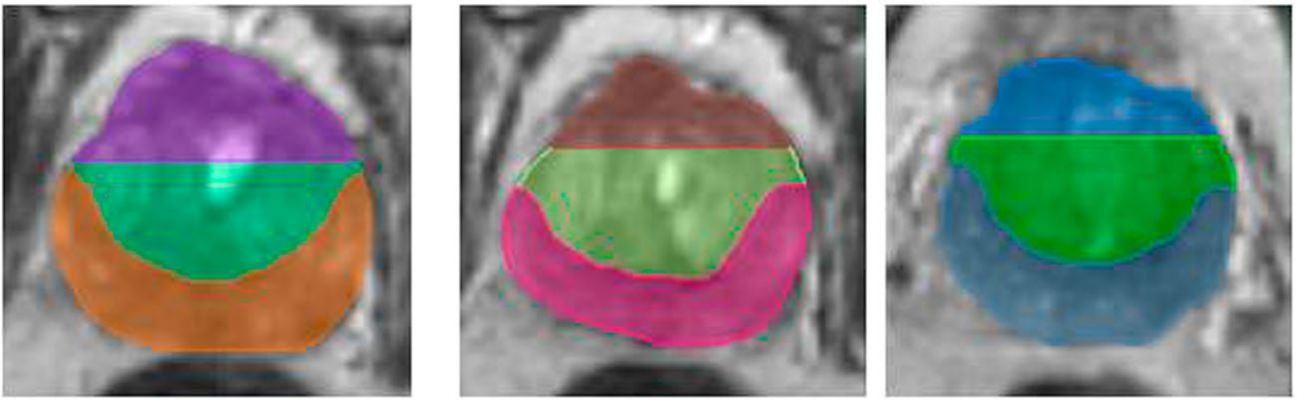
Prostate quadrants. The prostate and the Peripheral Zone (PZ) were manually contoured on the T2-weighted MRI in MIM. Transition Zone (TZ) contour was estimated algebraically by subtracting PZ from the prostate. The prostate is automatically segmented in three sections: apex, mid and base. 3 segments: PZ and two for TZ are automatically generated for each section, resulting in a 9-element 3D grid. Representative T2-weighted axial images in apex, mid and base with the corresponding grid are shown.

To identify and assign a level of risk of prostate cancer to various regions within the prostate, an automated software based on ADC was developed. The algorithm searched through ADC values to determine VOIs that matched the location of rpROIs. The prostate was divided in 9 quadrants (Fig. 4). The algorithm then determined ADC thresholds that *maximized* the sum of r, the Pearson Correlation coefficients between the nine pairs of fractions of rpROIs and ADC subvolumes in each quadrant, and ρ, Spearman’s ρ between VOIs and GSs. Three thresholds: D_HR_, D_IR,_ D_LR_ (D_HR_ < D_IR_ < D_LR_) were determined for both PZ and TZ, representing cut-points defining volumes related to areas at high (VOI_HR_), intermediate (VOI_IR_) and low risk (VOI_LR_) for cancer. Ultimately, three tables with different PZ and TZ cut-points for each set of risk volumes were created (Supplementary Tables S2–4). The results are summarized in Table 2. ADC thresholds for PZ/TZ, respectively are: high risk–900/800 µm^2^/s; intermediate risk–1100/850 µm^2^/s; low risk–1300/1050 µm^2^/s.

**Table 2:** Selected ADC thresholds and corresponding quantitative features.

Feature	High Risk	Intermediate Risk	Low Risk
ADC - PZ threshold	900	1100	1300
ADC - TZ threshold	800	850	1050
Min Volume	0.1	0.2	0.2
Spearman’s ρ	0.771	0.774	0.629
Average volume (cc)	0.364	0.733	2.296
# true positives	16	21	39
# false negatives	38	33	23
# false positives	4	3	3

**Figure 5:**
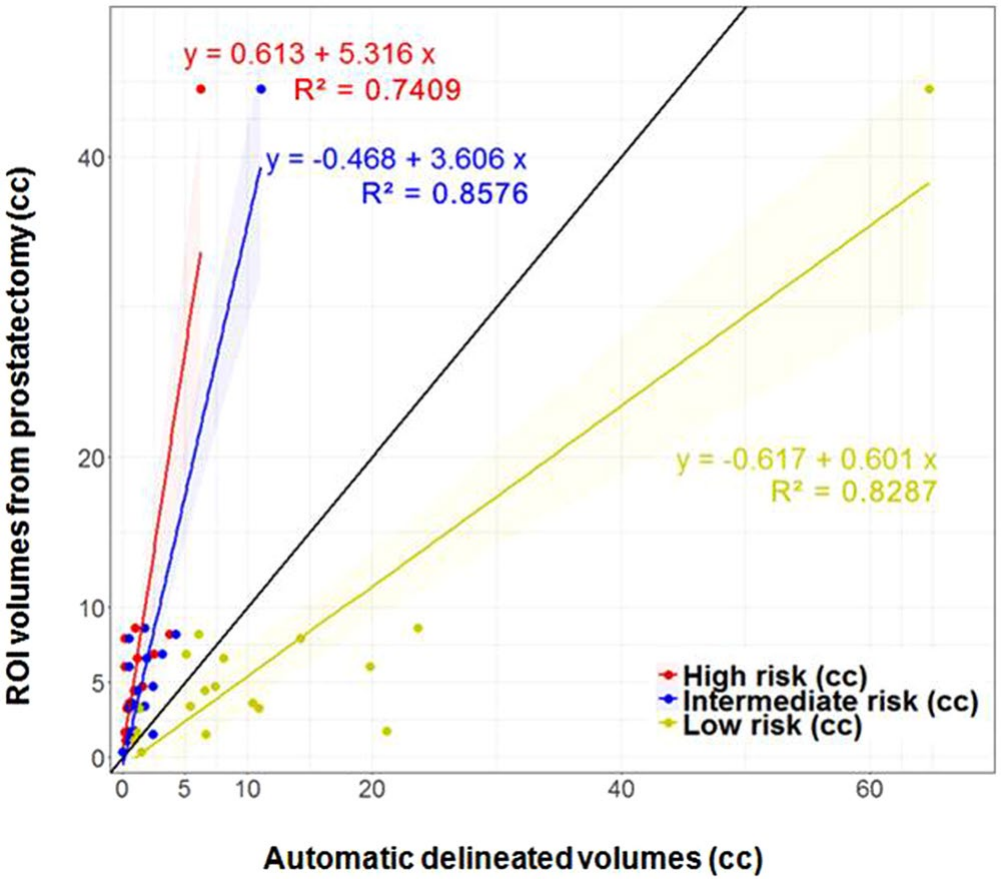
Correlation of prostatectomy ROIs and automatically delineated volumes, based on ADC thresholds. The black line is the unity line. The high and intermediate risk volumes are smaller than the prostatectomy ROIs. This is consistent with the notion that they are related to the more aggressive habitats of the tumor. The low volumes, by design, are supposed to map all tumor areas, including GS6. These volumes, however, appear to be larger by a factor of 1.4. If tissue-shrinkage factor (between 1.22 and 1.5) is considered, the unit line and regression line of the low risk volumes become co-linear.

VOI_HR_, VOI_IR_, and VOI_LR_ are illustrated in Fig. 1b. Based on the histopathology review, there is large inter- and intra-lesional heterogeneity. For instance, the anterior lesions in Fig. 1a vary in GS across the slices (GS = 6 in the apex and GS = 7 in mid and base). Note the excellent correlation between the red color intensities (habitats at high-risk) in Fig. 1b with the higher microscopic tumor grade. There are also areas of low (yellow color) and intermediate (blue color) risk in these lesions. VOI_HR_, VOI_IR_, and VOI_LR_ depict also the inter- and intra-lesional heterogeneity in the example in Fig. 2b. The regions in yellow depict areas of low risk and in general appear to be larger than the tumor on RP. This is illustrated on Fig. 5, where the relationship between rpROIs and VOI_HR_/VOI_IR_/VOI_LR_ are shown (Pearson correlation coefficient 0.86, 0.93 and 0.91, respectively). While all volumes were significantly correlated with the rpROIs (p < 0.0001), the high and intermediate risk volumes are smaller than the rpROIs. This is consistent with the notion that VOI_HR_ and VOI_IR_ are related to the more aggressive tumor habitats. The low-risk volumes, by design, are supposed to map all tumor areas, including GS6. These volumes appear to be larger by a factor of 1.4, that is consistent with reported RP tissue-shrinkage factor (1.22–1.5)^19^.

### Biopsy datasets

Twenty-eight target lesions were identified in the *targeted biopsy dataset* and 139 biopsies were analyzed in the *template biopsy datasets* (Table 1). Biopsy GS and PI-RADS were recorded for the locations of biopsies in the *targeted biopsy dataset* (tbROI). VOI_HR_, VOI_IR_ and VOI_LR_ were determined using D_HR_, D_IR_ and D_LR_ cut-points. Subvolumes, containing the tbROIs, were paired with the corresponding GS. For the patents in the *template biopsy dataset* only the highest GS from the biopsy session (6 to 18 biopsies) was available. The largest subvolume of VOI_HR_, VOI_IR_ and VOI_LR_ was *matched* with the patients’s GS.

**Figure 6:**
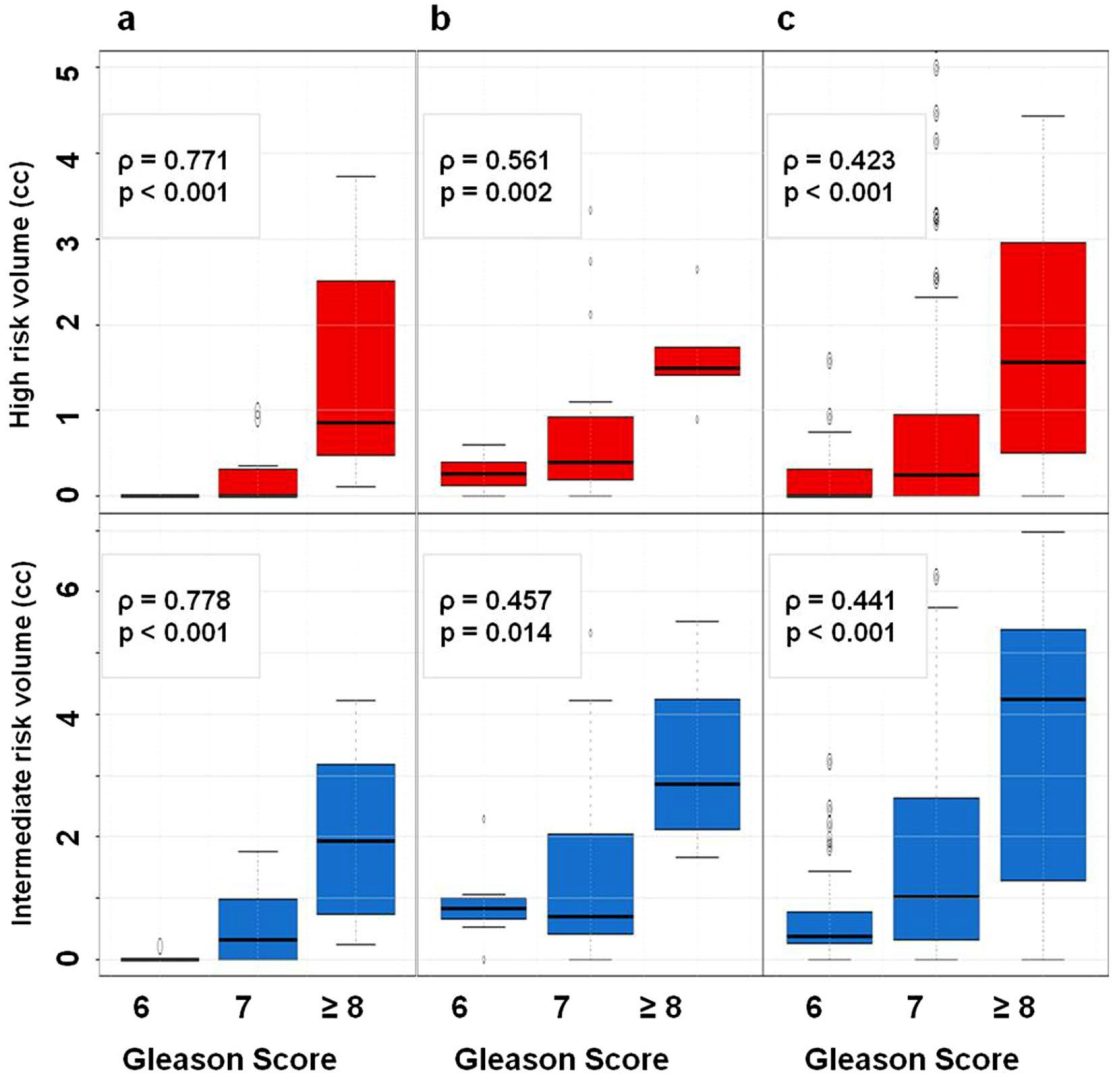
Box plots of distribution of volumes at high (top), intermediate (middle) and low (bottom) risk versus Gleason Score for patients from: (**a**) prostatectomy dataset, (**b**) targeted biopsy dataset; and (**c**) template biopsy dataset.

In Fig. 6 the associations of the sizes of VOI_HR_ and VOI_IR_ in all three datasets with GS are shown. All correlations, including VOI_LR_ (data not shown) were statistically significant.

**Figure 7:**
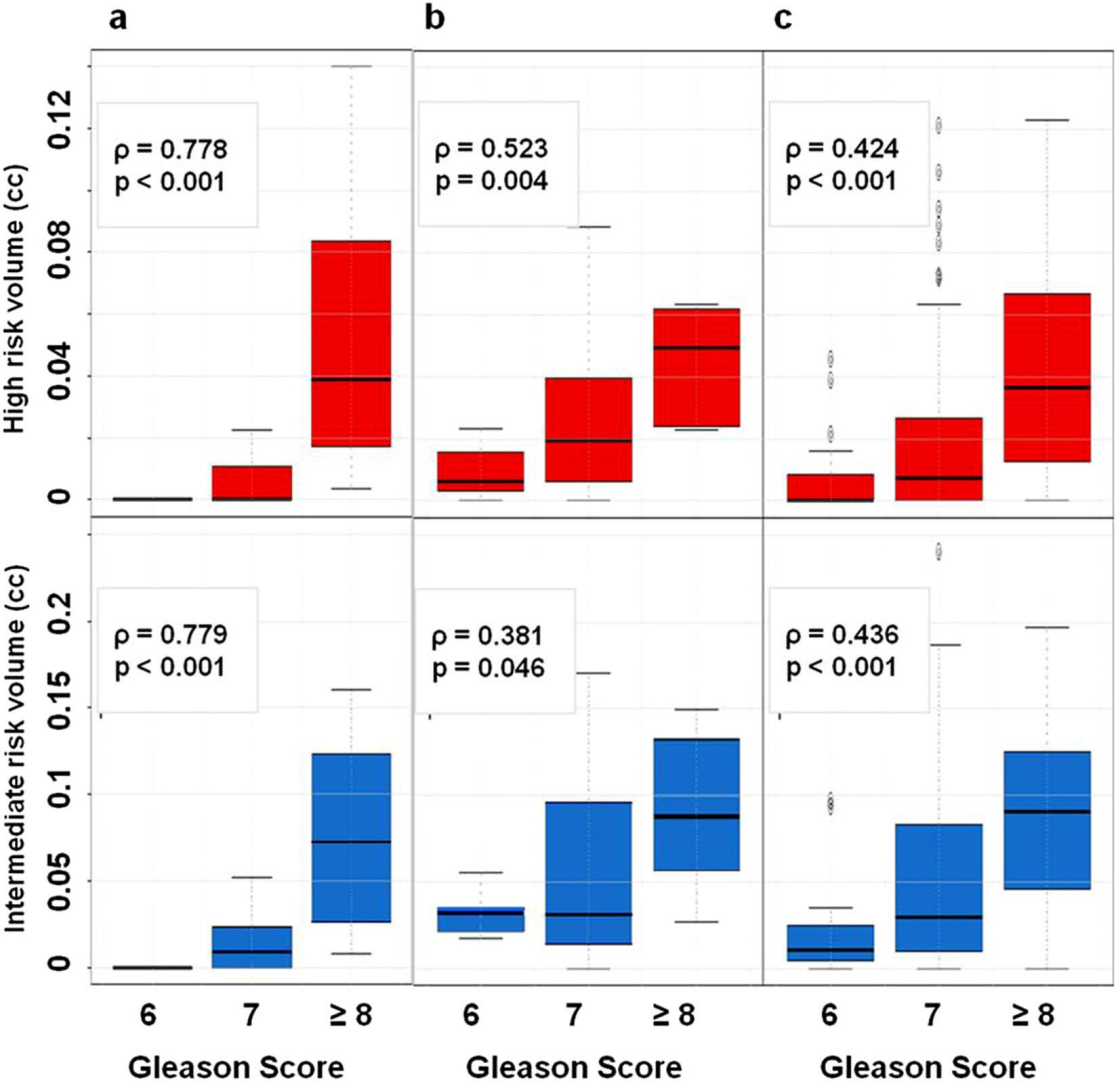
Box plots of identified relative volumes at high (red) and intermediate (blue) risk versus Gleason Score for patients of prostatectomy dataset (**a**), targeted biopsy dataset (**b**) and template biopsy dataset (**c**).

As expected, the highest correlations were in the *prostatectomy dataset*, as the tumors were most comprehensively annotated on histological slides and the thresholds were identified to maximize exactly these correlations. The correlations were decreasing with the reduction in precision of localization of the tumor lesions (from *targeted* to *template biopsy datasets*). The correlations remained very similar when the relative tumor volume (ratio between VOI_HR_/VOI_IR_/VOI_LR_ and prostate volume) was used (Fig. 7).

**Figure 8:**
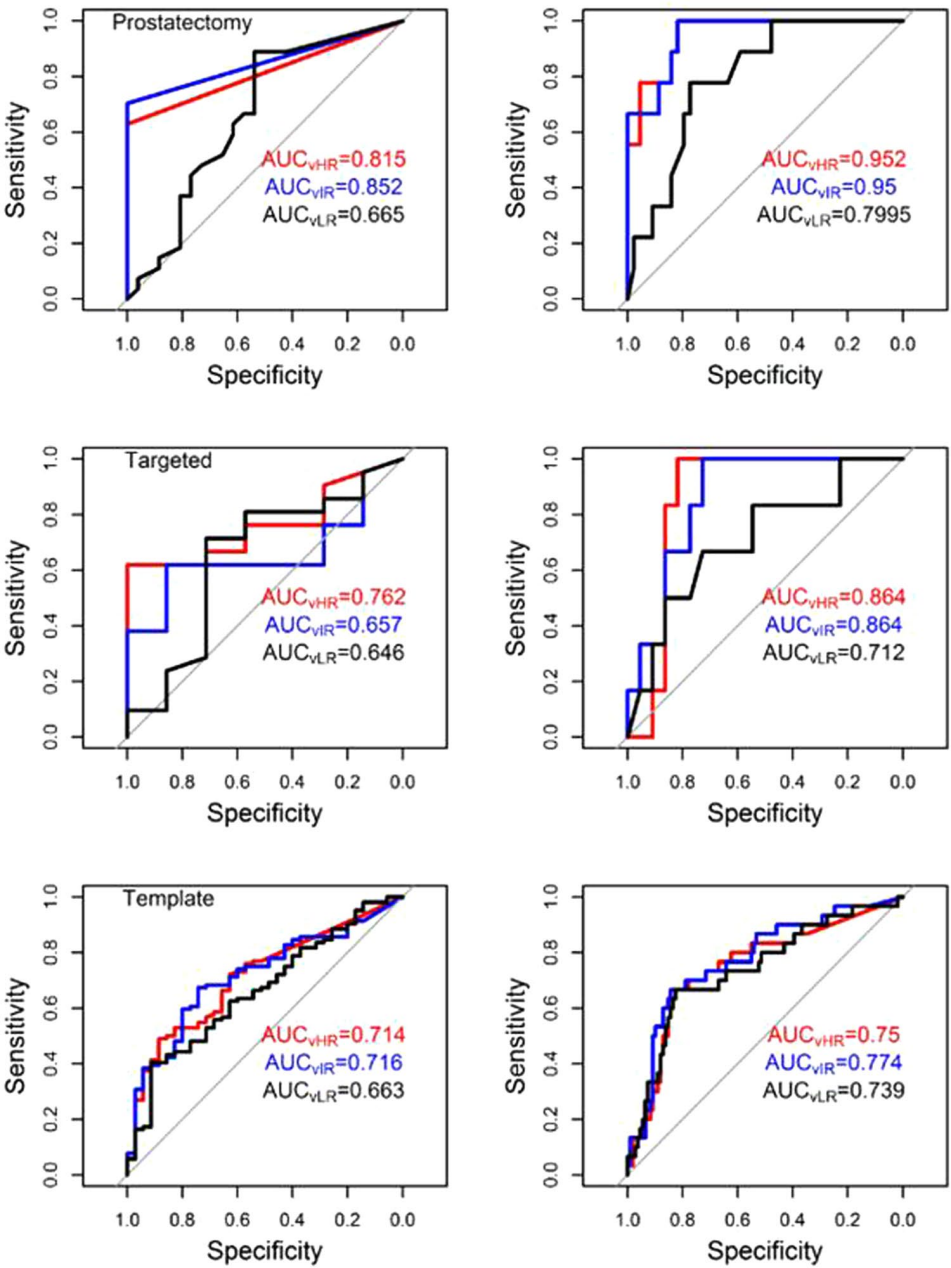
AUCs for identified volumes of high (red), intermediate (blue) and low (black) risk for discriminating (left) Gleason Score 6 vs 7 and higher lesions and (right) Gleason Score 6 and 7 vs 8 and higher lesions in prostatectomy dataset (**a**), targeted biopsy dataset (**b**) and template biopsy dataset (**c**). In the prostatectomy dataset, the *size* of the high risk VOIs (threshold 900/800 µm^2^/s, PZ/TZ) was strongly predictive for high grade tumors (GS 6,7 vs GS ≥ 8, AUC = 0.952); the *size* of the intermediate risk VOIs (threshold 1100/850 µm^2^/s, PZ/TZ) was strongly predictive for intermediate grade tumors (GS 6 vs GS ≥ 7, AUC = 0.852); and the low risk VOIs (threshold 1300/1050 µm^2^/s, PZ/TZ) coincided with all tumor nodules in RP.

Sensitivity, specificity, negative and positive predictive values and AUC for the VOIs for discriminating *(i)* GS6 versus 7+; and *(ii)* GS6,7 versus 8+ lesions are presented in Table 3 as well as the results for PI-RADS in the *targeted biopsy dataset*. AUC curves are shown in Fig. 8.

**Table 3:** Performance of ADC volumes and PI-RADS in relationship to Gleason Score.

*Radical prostatectomy*	Specificity	Sensitivity	NPV	PPV	AUC
**GS 6 vs GS 7–10**					
VOI_HR_*	1	0.63	0.722	1	0.8148
VOI_IR_**	1	0.704	0.765	1	0.8519
VOI_LR_^†^	0.538	0.889	0.824	0.667	0.6652
**GS 6–7 vs GS 8–10**					
VOI_HR_*	0.818	1	1	0.529	0.952
VOI_IR_**	0.818	1	1	0.529	0.9495
VOI_LR_^†^	0.773	0.778	0.944	0.412	0.7992
***Targeted Biopsies***					
**GS 6 vs GS 7–10**					
VOI_HR_*	1	0.619	0.467	1	0.7619
VOI_IR_**	0.857	0.619	0.429	1	0.8636
VOI_LR_^†^	0.714	0.714	0.455	0.882	0.6463
**GS 6–7 vs GS 8–10**					
VOI_HR_*	0.818	1	1	0.6	0.8636
VOI_IR_**	0.727	1	1	0.5	0.8636
VOI_LR_†	0.727	0.667	0.899	0.4	0.7121
***PI-RADS (Targeted Biopsies)***					
**GS 6 vs GS 7–10**					
PI-RADS 3–5	0.286	1	1	0.808	—
PI-RADS 4,5	0.429	0.905	0.6	0.826	—
**GS 6–7 vs GS 8–10**					
PI-RADS 3–5	0.091	1	1	0.231	—
PI-RADS 4,5	0.227	1	1	0.261	—
***Template Biopsies***					
**GS 6 vs GS 7–10**					
VOI_HR_*	0.866	0.49	0.369	0.927	0.713
VOI_IR_**	0.743	0.673	0.433	0.866	0.716
VOI_LR_^†^	0.914	0.404	0.34	0.933	0.663
**GS 6–7 vs GS 8–10**					
VOI_HR_*	0.826	0.667	0.9	0.513	0.75
VOI_IR_**	0.844	0.667	0.902	0.541	0.774
VOI_LR_^†^	0.826	0.667	0.9	0.513	0.74

*Abbreviations:* ADC = Apparent Diffusion Coefficient; AUC = Area under the curve; GS = Gleason Score; HR = High risk; IR = Intermediate risk; LR = Low risk; NPV = Negative Predictive Value; PPV = Positive Predictive Value; VOI = Volume of Interest.

^*^ADC < 900 (Peripheral Zone); ADC < 800 (Transition Zone).

^**^ADC < 1100 (Peripheral Zone); ADC < 850 (Transition Zone).

^†^ADC < 1300 (Peripheral Zone); ADC < 1050 (Transition Zone).

## Discussion

The use of mpMRI is rapidly gaining momentum in the management of prostate cancer. Currently, PI-RADS are the standard of care for identification of areas for targeted biopsy. The five-score system does not tap into the wealth of quantitative imaging information contained in the multiple sequences of mpMRI, nor does it elucidate intralesional spatial heterogeneity. In contrast, the proposed approach, based *only* on diffusion MRI, maps prostate tumor heterogeneity by assigning each pixel at three levels of risk for prostate cancer. This is the first study which identified ADC thresholds for volumes of reduced diffusion and associated the automatically identified volumes with cancer grade.

The size of the automatically delineated volumes in the prostate with restricted diffusion strongly correlate with cancer aggressiveness. The simplicity of the approach and strength of the determined relationships are quite revealing. The association of ADC volumes and GS (Figs 6 and 7), analogous to the correlation between rpROIs and GS (Fig. 3), is quite novel.

In comparison, when compared to high and intermediate ADC volumes in discriminating between GS6,7 and GS8+ lesions, PI-RADS assessments for sensitivity, specificity, NTV and PPV were lower (Table 3). In addition, PI-RADS contours do not depict the 3D tumor volume. While this may not be an objective of the radiologists/PI-RADS, areas of most aggressive disease need to be consistently and objectively identified in 3D for assigning biopsy targets or dose-boost areas in radiotherapy of the prostate.

This study has several limitations. First, this is a retrospective study with all inherent limitations of the design. Second, ADC thresholds were derived using a limited set of 18 RPs. The small sample size may present a problem for the generalizability of the identified thresholds, especially since the clinical characteristics in Table 1 indicate differences between the three datasets. For instance, based on PSA and T-stage, the patients in the ***prostatectomy*** and ***target biopsy datasets*** appear to be at higher risk relative to the patients in the ***template biopsy datasets***. Based on Gleason Score, however, the patients in the ***prostatectomy*** dataset are of lower risk: roughly 50% of the mapped 50 lesions were GS6. Gleason Score is the strongest, most reproducible predictor of clinical outcome and in this sense can be concluded that the prostatectomy patients are a lower risk cohort. Thus, the ADC thresholds, determined on this dataset warrant high sensitivity in detection of prostate cancer lesions. Third, only patients with proven cancer were included in the study as the diagnostic potential of the approach (i.e. cancer vs no cancer) was not a study objective. The application of the thresholds also requires contours of PZ and TZ. The use of a prostate atlas will decrease significantly the need of manual contouring^20^. Finally, the generalizability of the determined thresholds to other MRI sequences, vendors, magnetic field strengths and coils (endorectal vs body) should be investigated.

The ADC maps allow for: *(i)* better definition of biopsy targets for the identification of high grade disease and potentially the acquisition of tissue more representative of aggresiveness; and *(ii)* better definition of tumor volumes for any focal type of therapy, such as focused radiotherapy dose escalation. The identified ADC thresholds were recently integrated in Habitat Risk Score (HRS), a pixel by pixel ten-scale system that combines the quantitative Dynamic Contrast-Enhanced (DCE-)MRI and ADC^21^. HRS maps were used prospectively to guide radiotherapy (RT) boost volumes in a randomized Phase II clinical trial, comparing two methods of increasing dose to the mpMRI-defined tumor habitat region(s).

In conclusion, ADC thresholds for determining volumes at risk in the prostate are presented. The values can be utilized to generate 3D volumes or serve as guide for prostate evaluation.

## Methods

### MRI acquisition

mpMRIs of the prostate were acquired at 3 Tesla with a 32-channel phased array pelvis coil. Axial T2 weighted-MRI (T2w): 2D Fast spin-echo, 1.25 × 1.25 × 2.5 mm resolution; acquisition matrix = 256 × 256 and 72 slices; repetition time (TR)/echo time (TE)/Number of Excitations (NEX) = 5700/85/1; echo train length = 16; acquisition time = 4 minutes, flip angle 120°. DWI: single-shot echo-planar imaging utilizing the diffusion-module and fat-suppression pulses; 2.5 × 2.5 × 2.5 mm resolution; acquisition matrix = 128 × 128 and 36 slices; TR/TE/NEX 9500/53/1; a parallel imaging acceleration factor of two; acquisition time = 7 minutes. A minimum b value of 50 was chosen to avoid errors arising from perfusion-related intravoxel coherent motion, and a maximum of 1000 to avoid signal bias from the noise floor.

### Prostatectomy dataset

Patients that underwent RP during 2016 and had mpMRI on 3 T GE were identified from Urology Prostate database.

#### Mapping tumor lesions from radical prostatectomy on MRI

RP specimens were handled in accordance with a well-established procedure^16^. The radical prostatectomy (RP) specimens were first fixed overnight in ambient formalin without injection. The seminal vesicles were amputated and the prostate weight was recorded without seminal vesicles^16^. The prostates were inked for microscopic margin assessment and submitted entirely for histologic examination in regular size cassettes. Apex and base were cut at 5–7 mm into the prostate and submitted as perpendicular sections to the margin. The rest of the prostate was cut at 3–4 mm intervals perpendicular to the urethra and submitted as quadrants. All specimens were reviewed by a urologic pathologist (ONK) who was blinded to the imaging findings. Cancer was mapped on hematoxylin and eosin (H&E) stained slides (histopathology ROIs). Tumor nodules were considered spatially separate if they were at least 3 mm apart in a plane of section or at least 4 mm on consecutive sections^22^. Each tumor nodule was individually staged and graded according to the latest recommendations^17,18^. The prostate was then cut in quadrants, and tumor nodules contoured and graded by a urologic pathologist (ONK)^17,18^. The annotated slides were scanned and prostate quadrants were “stitched” into a pseudo whole-mount RP sample (Figs 1a and 2a).

Patient’s T2w and ADC were uploaded to a commercial image software package (MIM, Cleveland, OH). MIM is an image processing and analysis platform incorporating DICOM input/output, and includes fusion and contouring tools as well as an interface for user-defined algorithms. The goal was to ‘transfer’ the locations of the histopathology Regions of Interest (ROIs) from radical prostatectomy (rpROI) onto MRI. Correlation of MRI images with histopathologic whole-mount sections is challenging because of prostate deformation following prostatectomy and during fixation and processing^23^. The issues of one-to-one correspondence of MR images and histopathologic whole mount sections are well understood and acknowledged^23,24^. Following the approach, described in Wibmer *et al*.^5^ and using anatomical landmarks (urethra, ejaculatory ducts, hyperplastic nodules), freehand ROIs were drawn on the ADC maps and denoted rpROI. ROIs were drawn on all slices encompassing the lesion. Pathologist’s contours were then transferred on MRI using rpROIs^5,21^. The rpROIs volumes were estimated in MIM.

#### Determination of ADC thresholds for identifying prostate volumes at differing risk for cancer

**Figure 9:**
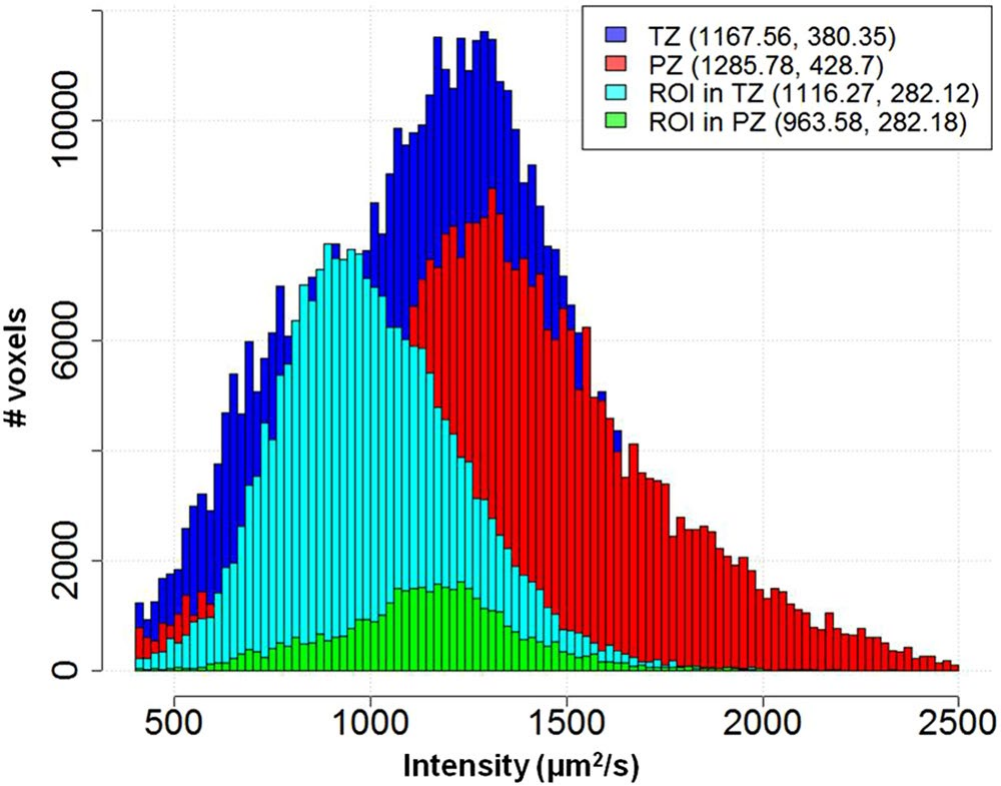
Histograms of ADC values in Transition Zone (TZ), Peripheral Zone (PZ) and Regions of Interest (ROI) in PZ and TZ mapped from histopathology. Note: TZ and PZ distributions are calculated after excluding the pixels within the ROIs. Mean and standard deviations are indicated in parentheses.

An automated algorithm for identification of optimal ADC thresholds for automatic delineation of prostatic lesions at differing risk for cancer was developed. Because of the differences in the signal intensities in PZ and TZ (Fig. 9), the algorithm determines zone-specific ADC thresholds. The gist of the method is iterating through ADC values (between 600 and 1300 µm^2^ at a step of 50 µm^2^); for each tested ADC threshold, the volumes, containing pixels with lower ADCs are evaluated in terms of overlap with rpROIs. The algorithm can be described as follows: Let N be the number of patients in the dataset. If there are I^m^ radical prostatectomy (RP) lesions for the m^th^ patient (m = 1, …, N), then ${{rpROI}}_{i}^{m}$ denotes the i^th^ lesion (i = 1, …, I^m^) with Gleason Score ${{GS}}_{i}^{m}$.

Three thresholds (D_HR_, D_IR_ and D_LR_, where D_HR_ < D_IR_ < D_LR_) are sought to identify the location and volumes at high (VOI_HR_), intermediate (VOI_IR_) and low risk (VOI_LR_) areas for cancer. The thresholds in ADC intensities are set separately for the peripheral zone (PZ) and transition zone (TZ). The routine developed in Java initially identifies D_HR_ by spanning ADC values between 700–1400 µm^2^/s in PZ and 600–1100 µm^2^/s in TZ by increments of 50 µm^2^/s. Pixels with intensities under 400 µm^2^/s were considered artefacts and not taken in account.

Let t be a pair of ADC thresholds in PZ and TZ; t = [t_PZ_, t_TZ_]. The volume ${V}_{t}^{m}$ in the m^th^ patient for the threshold t consists of voxels in PZ with ADC values < t_PZ_ and pixels in TZ with ADC values < t_TZ_. ${V}_{t}^{m}$ is composed of multiple disconnected volumes. For consistency, volumes less than β cc are removed using the “cleaning” utility in MIM. Assuming that there are J^m^ continuous volumes for the threshold t in the m^th^ patient, let ${V}_{{tj}}^{m}$ be the *j*^*th*^ volume (j = 1, …, J^m^). Each volume and rpROI were *matched* spatially using the 9 quadrants technique (Fig. 9).

**Figure 10:**
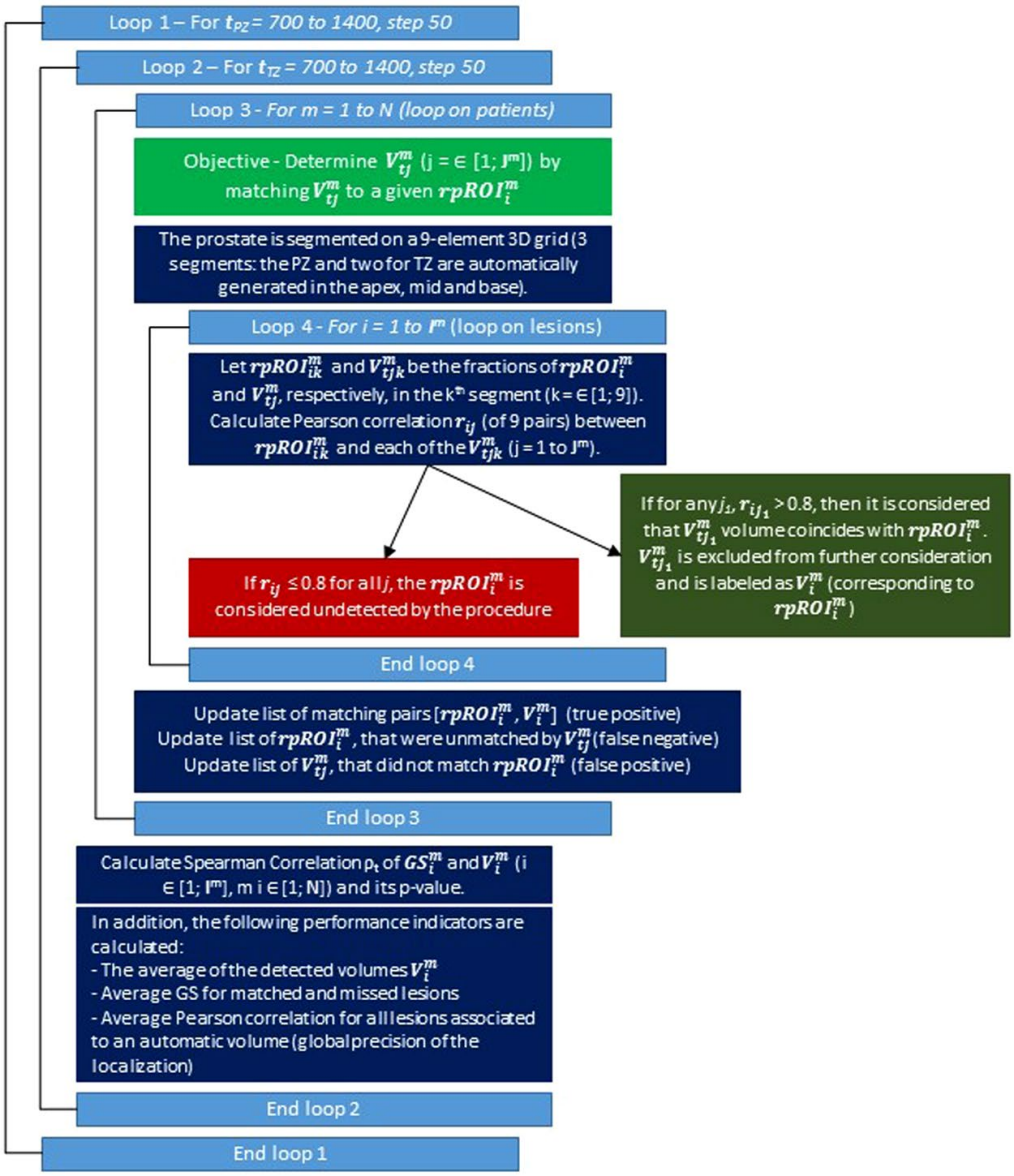
Graphical flowchart depicting search algorithm.

Figure 10 describes the 4 loops of the search algorithm. The thresholds in PZ and TZ for high risk are determined using β = 0.1 cc, and β = 0.2 cc was used for both the intermediate and low risk thresholds. The output for a subset of ADC values are shown in Supp. Tables 2–4 for high, intermediate and low risk, respectively. The PZ thresholds (Loop 1) are listed in the first row and TZ thresholds (Loop 2) – in the first column. For each threshold t = [t_PZ_, t_TZ_], Spearman correlations ρ_t_ between the size of the volumes and GS are displayed in the corresponding grid, highlighted with green (low) to red (high). Also listed are the p-value of ρ_t_, and the parameters a. to d. above. The pair [800, 900] results in the highest Spearman correlation 0.771, p-value ≪0.0001. The average detected volume is 0.364 cc; 18 (33.95%) of the rpROIs with average GS 7.89. This result indicates that the most aggressive lesions are captured with these thresholds. Finally, the average Pearson’s correlation between the fraction of volumes and RP lesions in the quadrants is reported.

Once thresholds D_HR_, D_IR,_ D_LR_ (D_HR_ < D_IR_ < D_LR_) were identified, areas at high (VOI_HR_), intermediate (VOI_IR_) and low risk (VOI_LR_) for cancer could be identified. The procedure was repeated for all patients in the *prostatectomy dataset*, resulting in VOIs that were paired with the GS of the corresponding rpROIs, and Spearman’s ρ between VOIs and GS was calculated.

### Biopsy datasets

The determined D_HR_, D_IR_ and D_LR_ were evaluated in the *target* and *template biopsy datasets*. Both cohorts of patients were identified from the Radiation Oncology Prostate database.

Patients in the *target biopsy dataset* participated in a contemporary radiotherapy clinical trial and underwent MRI-US biopsies as per protocol. MRI-US biopsies were carried out on Uronav (Invivo corp., Gainesville, FL). Prostate and suspicions lesions were contoured by an experienced fellowship-trained radiologist (FM) using the Dynacad (Invivo corp., Gainesville, FL) software, a general purpose 2D Computer Aided Diagnosis (CAD) program with the option of drawing ROIs to target with the biopsy. PI-RADS v.2^2^ were assigned to each biopsy target. A 3D transrectal US of the prostate was acquired prior to biopsy and the prostate volumes on US and MRI were co-registered using deformable fusion. Biopsy targets on MRI were transferred onto real-time ultrasound. According to the clinical trial protocol, only the designated target areas, usually 1 or 2, were biopsied. A maximum of three biopsy cores were acquired per target. Limited number of cores are acquired in order to decrease the risk for bleeding, as the patients are planned to start radiotherapy within a month from the biopsy session. The biopsy tissue was reviewed by an urologic pathologist (ONK).

The locations of biopsies with GS ≥ 6 (tbROI) were transferred to MRI in MIM, using the biopsy needle tracks in Uronav. Biopsy GS and PI-RADS were recorded for each tbROI. VOI_HR_, VOI_IR_ and VOI_LR_ were determined using D_HR_, D_IR_ and D_LR_ cut-points. Subvolumes, containing the tbROIs, were paired with the corresponding GS. As 1 to 3 biopsies were acquired per target, the highest GS is used for ADC volume correlations. VOI_HR_, VOI_IR_, and VOI_LR_ are calculated as sets of pixels with ADC < D_HR_, D_IR,_ and D_LR,_ respectively. VOI_HR_, VOI_IR_, and VOI_LR_ consist typically of multiple discontinuous volumes across the prostate. Small volumes (<0.1 cc) are “cleaned” and the remaining continuous volumes are sorted by size and labeled. For instance, let VOI_HR_ consist of three subvolumes. These volumes can be visualized in MIM and the one containing the biopsy needle is selected to be paired with the biopsy GS.

For the patents in the *template biopsy dataset* only the highest GS from the biopsy session (6 to 18 biopsies) was available. The largest subvolume of VOI_HR_, VOI_IR_ and VOI_LR_ was *matched* with the patients’s GS.

### Statistical analysis

For each of the three cohorts, Spearman’s ρ was calculated between: *(i)* the size of the VOIs of “low” ADC, and *(ii)* the GS of the corresponding RP lesion/biopsy. Sensitivity, specificity, negative and positive predicative values for GS were calculated. For comparison, the same assessments were carried out for PI-RADS in the *targeted biopsy dataset* (PI-RADS were not available for the majority of the patients in the *prostatectomy* and *template biopsy datasets*).

The area under the curve (AUC) of the receiver operating characteristics (ROC) analysis was computed using a logistic regression model. Statistical analysis was performed using a statistics software package (R, http://www.R-project.org/). All tests were two-sided. Significance was set at *p-value* < 0.05.

## Electronic supplementary material

Supplementary Tables

## References

[1] Barentsz JO (2012). ESUR prostate MR guidelines 2012. Eur Radiol.

[2] Hassanzadeh E (2017). Prostate imaging reporting and data system version 2 (PI-RADSv2): a pictorial review. Abdom Radiol (NY).

[3] Khalvati F, Wong A, Haider MA (2015). Automated prostate cancer detection via comprehensive multi-parametric magnetic resonance imaging texture feature models. BMC Med Imaging.

[4] Cameron A, Khalvati F, Haider MA, Wong A (2016). MAPS: A Quantitative Radiomics Approach for Prostate Cancer Detection. IEEE Trans Biomed Eng.

[5] Wibmer A (2015). Haralick texture analysis of prostate MRI: utility for differentiating non-cancerous prostate from prostate cancer and differentiating prostate cancers with different Gleason scores. Eur Radiol.

[6] Fehr D (2015). Automatic classification of prostate cancer Gleason scores from multiparametric magnetic resonance images. Proc Natl Acad Sci USA.

[7] Litjens GJS (2016). Computer-extracted Features Can Distinguish Noncancerous Confounding Disease from Prostatic Adenocarcinoma at Multiparametric MR Imaging. Radiology.

[8] Sandler, K. *et al*. Multiparametric-MRI and targeted biopsies in the management of prostate cancer patients on active surveillance. *Front Oncol* **5**, 10.3389/fonc.2015.00004 (2015). 10.3389/fonc.2015.00004 PMC4306300 25674540

[9] Somford DM (2012). Initial experience with identifying high-grade prostate cancer using diffusion-weighted MR imaging (DWI) in patients with a Gleason score </ = 3 + 3 = 6 upon schematic TRUS-guided biopsy: a radical prostatectomy correlated series. Invest Radiol.

[10] Litjens GJ, Hambrock T, Hulsbergen-van de Kaa C, Barentsz JO, Huisman HJ (2012). Interpatient variation in normal peripheral zone apparent diffusion coefficient: effect on the prediction of prostate cancer aggressiveness. Radiology.

[11] Hoeks CM (2013). Diffusion-weighted magnetic resonance imaging in the prostate transition zone: histopathological validation using magnetic resonance-guided biopsy specimens. Invest Radiol.

[12] Hambrock T (2011). Relationship between Apparent Diffusion Coefficients at 3.0-T MR Imaging and Gleason Grade in Peripheral Zone Prostate Cancer. Radiology.

[13] Hambrock T (2012). Prospective assessment of prostate cancer aggressiveness using 3-T diffusion-weighted magnetic resonance imaging-guided biopsies versus a systematic 10-core transrectal ultrasound prostate biopsy cohort. European urology.

[14] Bittencourt LK, Barentsz JO, de Miranda LC, Gasparetto EL (2012). Prostate MRI: diffusion-weighted imaging at 1.5T correlates better with prostatectomy Gleason Grades than TRUS-guided biopsies in peripheral zone tumours. Eur Radiol.

[15] Tschudi Y (2016). Association of Prostate Volumes with Restricted Diffusion and Prostate Cancer Aggressiveness. Med Phys.

[16] Tjionas GA (2015). Average Weight of Seminal Vesicles: An Adjustment Factor for Radical Prostatectomy Specimens Weighed With Seminal Vesicles. Int J Surg Pathol.

[17] Kryvenko ON, Epstein JI (2016). Prostate Cancer Grading: A Decade After the 2005 Modified Gleason Grading System. Arch Pathol Lab Med.

[18] Kryvenko ON, Epstein JI (2016). Changes in prostate cancer grading: Including a new patient-centric grading system. Prostate.

[19] Schned AR (1996). Tissue-shrinkage correction factor in the calculation of prostate cancer volume. Am J Surg Pathol.

[20] Nelson AS (2015). Evaluation of An Atlas-Based Segmentation Method for Prostate and Peripheral Zone Regions On MRI. Med Phys.

[21] Stoyanova Radka, Chinea Felix, Kwon Deukwoo, Reis Isildinha M., Tschudi Yohann, Parra Nestor A., Breto Adrian L., Padgett Kyle R., Pra Alan Dal, Abramowitz Matthew C., Kryvenko Oleksandr N., Punnen Sanoj, Pollack Alan (2018). An Automated Multiparametric MRI Quantitative Imaging Prostate Habitat Risk Scoring System for Defining External Beam Radiation Therapy Boost Volumes. International Journal of Radiation Oncology*Biology*Physics.

[22] Kryvenko ON, Carter HB, Trock BJ, Epstein JI (2014). Biopsy criteria for determining appropriateness for active surveillance in the modern era. Urology.

[23] Turkbey B (2010). Prostate cancer: value of multiparametric MR imaging at 3 T for detection–histopathologic correlation. Radiology.

[24] Futterer JJ (2006). Prostate cancer localization with dynamic contrast-enhanced MR imaging and proton MR spectroscopic imaging. Radiology.

