# Supplementary material for: Automatic Detection of Prostate Tumor Habitats using Diffusion MRI
Source: Sci Rep. 2018 Nov 14;8:16801. doi: 10.1038/s41598-018-34916-4 (PMC6235961; doi:10.1038/s41598-018-34916-4)
Supplement: Supplementary file 1 — Supplementary Tables [file 41598_2018_34916_MOESM1_ESM.pdf]

Automatic Detection of Prostate Tumor Habitats using Diffusion MRI

Yohann Tschudi, Ph.D., Alan Pollack, M.D., Ph.D., Sanoj Punnen, M.D., John C. Ford, Ph.D., Yu-Cherng Chang, B.S., Nachiketh Soodana-Prakash, M.D., Adrian L. Breto, B.S., Deukwoo Kwon, Ph.D., Felipe Munera, M.D., Matthew C. Abramowitz, M.D., Oleksandr N. Kryvenko, M.D., Radka Stoyanova, Ph.D.

Supplementary Table 1. Summary of radical prostatectomy ROIs.

Patient	ROI	Location	Volume	GS pattern	GS
1	1	TZ	0.38	3+3	6
1	2	TZ	0	3+3	6
2	1	TZ	3.61	3+4	7
2	2	PZ	0.31	3+3	6
2	3	PZ	3.46	3+4	7
2	4	PZ	0.51	3+3	6
3	1	PZ	1.2	4+5	9
3	2	PZ	1.29	3+4	7
3	3	TZ	0.18	3+3	6
3	4	TZ	0.69	3+4	7
4	1	TZ	43.84	5+5	10
4	2	PZ	0.68	3+3	6
5	1	TZ	3.57	3+4	7
5	2	PZ	0.34	3+3	6
5	3	TZ	0.18	3+3	6
5	4	TZ	0	3+4	7
5	5	TZ	0.37	3+4	7
5	6	PZ	0.06	3+3	6
6	1	TZ	6.37	4+5	9
6	2	TZ	0.29	3+3	6
6	3	PZ	0.263	3+4	7
7	1	TZ	2.48	4+5	9
7	2	PZ	0.87	3+4	7
7	3	PZ	0.64	3+4	7
8	1	TZ	2.16	3+4	7
8	2	PZ	0.54	3+3	6
8	3	PZ	0.36	3+3	6
9	1	TZ	6.63	4+5	9
10	1	TZ	7.42	3+4	7
10	2	TZ	0.27	3+3	6
10	3	PZ	0.68	3+3	6
10	4	PZ	0.27	3+3	6

Patient	ROI	Location	Volume	GS pattern	GS
11	1	PZ	1.98	3+4	7
11	2	PZ	0.63	3+4	7
11	3	TZ	0.39	3+3	6
11	4	PZ	0.2	3+3	6
11	5	TZ	0.07	3+3	6
12	1	PZ	0.59	3+4	7
12	2	PZ	0.31	3+3	6
12	3	PZ	0.21	3+3	6
13	1	TZ	4.72	4+5	9
14	1	PZ	0.76	3+4	7
14	2	TZ	0.75	3+3	6
14	3	PZ	0	3+3	6
14	4	PZ	0.23	3+3	6
15	1	PZ	1.44	5+4	9
15	2	TZ	0.22	3+3	6
16	1	TZ	7.83	4+5	9
16	2	PZ	0.35	3+4	7
17	1	TZ	2.55	3+4	7
17	2	TZ	0.51	3+3	6
17	3	PZ	0.22	3+3	6
18	1	PZ	1.08	4+4	8

<u>Supplementary Table S2.</u> ADC thresholds for determining volumes at high risk (PZ/TZ=900/800 μm²/s)

TZ \ PZ	Statistics	700	750	800	850	900	950	1000
600	Spearman's ρ	0.442	0.504	0.526	0.536	0.583	0.516	0.478
	p-value	9.28E-04	1.20E-04	5.13E-05	3.51E-05	4.65E-06	7.60E-05	2.95E-04
	|Volume| (cc)	0.038	0.044	0.058	0.069	0.085	0.108	0.142
	True Positive/False Negative/False Positive	5/48/0	5/48/1	10/43/1	11/42/1	11/42/2	11/42/5	12/42/7
	|Pearson's r|	0.852	0.851	0.889	0.882	0.88	0.91	0.9
	|GS True Positive|/|GS False Positive|	8/6.73	8/6.73	7.3/6.74	7.27/6.74	7.27/6.74	7/6.81	7/6.81
650	Spearman's ρ	0.502	0.555	0.577	0.591	0.631	0.571	0.494
	p-value	1.30E-04	1.60E-05	6.08E-06	3.13E-06	3.99E-07	8.05E-06	1.68E-04
	|Volume| (cc)	0.063	0.069	0.083	0.094	0.110	0.133	0.166
	True Positive/False Negative/False Positive	5/48/1	5/48/2	10/43/2	11/42/2	12/41/2	12/41/5	12/42/8
	|Pearson's r|	0.896	0.896	0.912	0.904	0.887	0.907	0.909
	|GS True Positive|/|GS False Positive|	8/6.73	8/6.73	7.3/6.74	7.27/6.74	7.42/6.68	7.17/6.76	7/6.81
700	Spearman's ρ		0.608	0.641	0.641	0.688	0.625	0.559
	p-value		1.38E-06	2.31E-07	2.31E-07	1.26E-08	5.60E-07	1.33E-05
	|Volume| (cc)		0.118	0.133	0.143	0.160	0.184	0.217
	True Positive/False Negative/False Positive		5/48/4	12/41/4	12/41/4	14/39/3	14/39/5	15/39/6
	|Pearson's r|		0.899	0.884	0.889	0.871	0.895	0.884
	|GS True Positive|/|GS False Positive|		8.6/6.67	7.5/6.66	7.5/6.66	7.71/6.54	7.5/6.62	7.47/6.62
750	Spearman's ρ			0.593	0.598	0.651	0.621	0.574
	p-value			2.92E-06	2.31E-06	1.30E-07	7.19E-07	6.96E-06
	|Volume| (cc)			0.208	0.219	0.235	0.258	0.292
	True Positive/False Negative/False Positive			18/36/3	17/37/4	17/37/5	19/35/6	20/35/8
	|Pearson's r|			0.87	0.884	0.888	0.887	0.881
	|GS True Positive|/|GS False Positive|			7.72/6.47	7.71/6.51	7.71/6.51	7.58/6.51	7.55/6.51
800	Spearman's ρ				0.727	0.771	0.737	0.659
	p-value				6.88E-10	1.49E-11	3.09E-10	8.20E-08
	|Volume| (cc)				0.350	0.367	0.391	0.427
	True Positive/False Negative/False Positive				18/36/2	17/37/4	20/34/5	20/35/8
	|Pearson's r|				0.889	0.903	0.896	0.893
	|GS True Positive|/|GS False Positive|				7.72/6.47	7.71/6.51	7.6/6.47	7.55/6.51
850	Spearman's ρ					0.745	0.701	0.666
	p-value					1.54E-10	5.06E-09	5.34E-08
	|Volume| (cc)					0.524	0.549	0.584
	True Positive/False Negative/False Positive					19/35/4	23/33/5	21/34/9
	|Pearson's r|					0.904	0.89	0.901
	|GS True Positive|/|GS False Positive|					7.79/6.4	7.65/6.39	7.62/6.44
900	Spearman's ρ						0.684	0.667
	p-value						1.63E-08	5.07E-08
	|Volume| (cc)						0.727	0.765
	True Positive/False Negative/False Positive						24/33/5	24/32/6
	|Pearson's r|						0.916	0.912
	|GS True Positive|/|GS False Positive|						7.67/6.42	7.62/6.41

The following six parameters were calculated for each tested pair of PZ/TZ thresholds:

1. Spearman correlation coefficient ρ between the size of the resultant VOIs and GS of the corresponding RP lesion;
2. P-value of the Spearman's ρ;
3. Average volume of VOIs;
4. True positive = VOI is matching RP lesion; False negative = there is no VOI that matches the RP lesion; False positive = there is no RP lesion that matches the VOI;
5. Pearson correlation coefficient r between the fractions of VOIs and RP lesion in the quadrants;
6. Average GS of True Positive VOIs / Average GS of False Positive VOIs

<u>Supplementary Table S3.</u> ADC thresholds for determining volumes at intermediate risk (PZ/TZ=1100/850 $\mu m^2/s$)

TZ \ PZ	Statistics	900	950	1000	1050	1100	1150	1200
800	Spearman's ρ	0.671	0.714	0.686	0.704	0.761	0.704	0.697
	p-value	3.90E-08	1.94E-09	1.39E-08	4.16E-09	3.83E-11	4.05E-09	6.76E-09
	|Volume| (cc)	0.356	0.378	0.407	0.438	0.494	0.607	0.795
	True Positive/False Negative/False Positive	17/37/1	18/36/2	18/36/3	20/35/2	23/31/2	24/30/7	24/31/9
	|Pearson's r|	0.899	0.894	0.895	0.900	0.892	0.909	0.928
	|GS True Positive|/|GS False Positive|	7.71/6.3	7.67/6.31	7.67/6.26	7.55/6.28	7.57/6.59	7.42/6.52	7.21/6.62
850	Spearman's ρ	0.714	0.755	0.729	0.750	0.778	0.722	0.703
	p-value	1.94E-09	6.42E-11	6.11E-10	1.06E-10	7.13E-12	1.06E-09	4.24E-09
	|Volume| (cc)	0.510	0.532	0.561	0.592	0.649	0.767	0.952
	True Positive/False Negative/False Positive	19/35/0	19/35/1	18/36/3	20/34/2	24/30/2	26/29/7	27/28/8
	|Pearson's r|	0.901	0.904	0.915	0.917	0.900	0.909	0.917
	|GS True Positive|/|GS False Positive|	7.79/6.3	7.79/6.35	7.78/6.3	7.65/6.52	7.62/6.61	7.54/6.53	7.3/6.62
900	Spearman's ρ		0.769	0.745	0.766	0.738	0.747	0.710
	p-value		1.71E-11	1.54E-10	2.27E-11	2.83E-10	1.28E-10	2.63E-09
	|Volume| (cc)		0.712	0.744	0.777	0.840	0.956	1.133
	True Positive/False Negative/False Positive		20/35/2	19/35/3	22/33/2	24/30/5	27/28/7	28/27/8
	|Pearson's r|		0.922	0.927	0.915	0.914	0.925	0.925
	|GS True Positive|/|GS False Positive|		7.8/6.36	7.79/6.32	7.64/6.32	7.67/6.64	7.48/6.57	7.29/6.57
950	Spearman's ρ			0.740	0.723	0.701	0.733	0.698
	p-value			2.46E-10	9.45E-10	5.13E-09	4.52E-10	6.22E-09
	|Volume| (cc)			0.929	0.964	1.028	1.147	1.322
	True Positive/False Negative/False Positive			22/33/3	22/33/4	25/29/5	29/26/5	31/24/6
	|Pearson's r|			0.920	0.936	0.923	0.932	0.926
	|GS True Positive|/|GS False Positive|			7.59/6.27	7.59/6.3	7.6/6.55	7.41/6.6	7.26/6.6
1000	Spearman's ρ				0.708	0.670	0.732	0.694
	p-value				2.97E-09	4.17E-08	4.64E-10	8.24E-09
	|Volume| (cc)				1.155	1.221	1.329	1.506
	True Positive/False Negative/False Positive				24/31/3	28/27/4	30/26/4	33/23/3
	|Pearson's r|				0.940	0.930	0.933	0.921
	|GS True Positive|/|GS False Positive|				7.42/6.33	7.43/6.62	7.37/6.54	7.24/6.63

The following six parameters were calculated for each tested pair of PZ/TZ thresholds:

1. Spearman correlation coefficient ρ between the size of the resultant VOIs and GS of the corresponding RP lesion;
2. P-value of the Spearman's ρ;
3. Average volume of VOIs;
4. True positive = VOI is matching RP lesion; False negative = there is no VOI that matches the RP lesion; False positive = there is no RP lesion that matches the VOI;
5. Pearson correlation coefficient r between the fractions of VOIs and RP lesion in the quadrants;
6. Average GS of True Positive VOIs / Average GS of False Positive VOIs

Supplementary Table S4. ADC thresholds for determining volumes at low risk (PZ/TZ=1300/1050 $\mu m^2/s$)

TZ \ PZ	Statistics	1100	1150	1200	1250	1300	1350	1400
1000	Spearman's ρ	0.670	0.732	0.694	0.713	0.641	0.632	0.671
	p-value	4.17E-08	4.64E-10	8.24E-09	2.14E-09	2.29E-07	3.78E-07	3.88E-08
	|Volume| (cc)	1.221	1.329	1.506	1.738	2.075	2.404	2.725
	True Positive/False Negative/False Positive	30/25/2	34/23/4	37/21/3	32/24/4	35/25/4	32/26/5	25/30/6
	|Pearson's r|	0.911	0.900	0.893	0.904	0.876	0.871	0.860
	|GS True Positive|/|GS False Positive|	7.37/6.28	7.29/6.26	7.27/6.19	7.31/6.25	7.09/6.6	7.06/6.58	7.24/6.57
1050	Spearman's ρ	0.650	0.678	0.677	0.667	0.629	0.618	0.666
	p-value	1.43E-07	2.52E-08	2.67E-08	5.06E-08	4.64E-07	8.07E-07	5.21E-08
	|Volume| (cc)	1.438	1.552	1.728	2.014	2.296	2.616	2.938
	True Positive/False Negative/False Positive	33/24/3	40/19/4	42/18/3	34/23/2	39/23/3	36/24/5	27/28/6
	|Pearson's r|	0.898	0.892	0.884	0.885	0.865	0.862	0.847
	|GS True Positive|/|GS False Positive|	7.3/6.25	7.15/6.26	7.17/6.17	7.18/6.3	7.08/6.52	6.97/6.62	7.19/6.57
1100	Spearman's ρ		0.661	0.663	0.631	0.616	0.638	0.673
	p-value		7.21E-08	6.35E-08	4.02E-07	9.00E-07	2.73E-07	3.49E-08
	|Volume| (cc)		1.844	2.024	2.267	2.552	2.870	3.190
	True Positive/False Negative/False Positive		40/19/3	40/19/3	35/22/2	35/24/3	33/24/4	27/27/5
	|Pearson's r|		0.881	0.887	0.881	0.874	0.871	0.846
	|GS True Positive|/|GS False Positive|		7.08/6.26	7.1/6.26	7.2/6.23	7.14/6.54	7.12/6.5	7.15/6.63
1150	Spearman's ρ			0.685	0.643	0.638	0.661	0.673
	p-value			1.56E-08	2.12E-07	2.79E-07	7.16E-08	3.49E-08
	|Volume| (cc)			2.364	2.600	2.898	3.199	3.519
	True Positive/False Negative/False Positive			37/21/3	34/21/2	34/25/4	29/28/6	27/27/7
	|Pearson's r|			0.905	0.898	0.872	0.886	0.851
	|GS True Positive|/|GS False Positive|			7.14/6.29	7.21/6.24	7.15/6.56	7.17/6.54	7.15/6.63
1200	Spearman's ρ				0.642	0.610	0.651	0.659
	p-value				2.17E-07	1.27E-06	1.31E-07	8.10E-08
	|Volume| (cc)				2.963	3.260	3.573	3.898
	True Positive/False Negative/False Positive				35/20/2	38/20/4	30/27/6	26/27/6
	|Pearson's r|				0.907	0.859	0.873	0.865
	|GS True Positive|/|GS False Positive|				7.17/6.25	7.05/6.5	7.13/6.56	7.12/6.59

The following six parameters were calculated for each tested pair of PZ/TZ thresholds:

1. Spearman correlation coefficient ρ between the size of the resultant VOIs and GS of the corresponding RP lesion;
2. P-value of the Spearman's ρ;
3. Average volume of VOIs;
4. True positive = VOI is matching RP lesion; False negative = there is no VOI that matches the RP lesion; False positive = there is no RP lesion that matches the VOI;
5. Pearson correlation coefficient r between the fractions of VOIs and RP lesion in the quadrants;
6. Average GS of True Positive VOIs / Average GS of False Positive VOIs
